# Astrocytes from cortex and striatum show differential responses to mitochondrial toxin and BDNF: implications for protection of striatal neurons expressing mutant huntingtin

**DOI:** 10.1186/s12974-020-01965-4

**Published:** 2020-10-06

**Authors:** Julieta Saba, Federico López Couselo, Juan Turati, Lila Carniglia, Daniela Durand, Andrea de Laurentiis, Mercedes Lasaga, Carla Caruso

**Affiliations:** 1 Instituto de Investigaciones Biomédicas (INBIOMED), UBA-CONICET, Paraguay 2155, Facultad de Medicina, Universidad de Buenos Aires, Buenos Aires, Argentina; 2 Centro de Estudios Farmacológicos y Botánicos (CEFYBO). UBA-CONICET, Paraguay 2155, Facultad de Medicina, Universidad de Buenos Aires, Buenos Aires, Argentina

**Keywords:** BDNF, Striatal astrocytes, Cortical astrocytes, 3-Nitropropionic acid, Huntington’s disease, Glutamate transporters, TNF-α, TGF-β, Astrocyte-conditioned medium, ST14A-Q120 striatal neurons

## Abstract

**Background:**

Evidence shows significant heterogeneity in astrocyte gene expression and function. We previously demonstrated that brain-derived neurotrophic factor (BDNF) exerts protective effects on whole brain primary cultured rat astrocytes treated with 3-nitropropionic acid (3NP), a mitochondrial toxin widely used as an in vitro model of Huntington’s disease (HD). Therefore, we now investigated 3NP and BDNF effects on astrocytes from two areas involved in HD: the striatum and the entire cortex, and their involvement in neuron survival.

**Methods:**

We prepared primary cultured rat cortical or striatal astrocytes and treated them with BDNF and/or 3NP for 24 h. In these cells, we assessed expression of astrocyte markers, BDNF receptor, and glutamate transporters, and cytokine release. We prepared astrocyte-conditioned medium (ACM) from cortical and striatal astrocytes and tested its effect on a cellular model of HD.

**Results:**

BDNF protected astrocytes from 3NP-induced death, increased expression of its own receptor, and activation of ERK in both cortical and striatal astrocytes. However, BDNF modulated glutamate transporter expression differently by increasing GLT1 and GLAST expression in cortical astrocytes but only GLT1 expression in striatal astrocytes. Striatal astrocytes released higher amounts of tumor necrosis factor-α than cortical astrocytes in response to 3NP but BDNF decreased this effect in both populations. 3NP decreased transforming growth factor-β release only in cortical astrocytes, whereas BDNF treatment increased its release only in striatal astrocytes. Finally, we evaluated ACM effect on a cellular model of HD: the rat striatal neuron cell line ST14A expressing mutant human huntingtin (Q120) or in ST14A cells expressing normal human huntingtin (Q15). Neither striatal nor cortical ACM modified the viability of Q15 cells. Only ACM from striatal astrocytes treated with BDNF and ACM from 3NP + BDNF-treated striatal astrocytes protected Q120 cells, whereas ACM from cortical astrocytes did not.

**Conclusions:**

Data suggest that cortical and striatal astrocytes respond differently to mitochondrial toxin 3NP and BDNF. Moreover, striatal astrocytes secrete soluble neuroprotective factors in response to BDNF that selectively protect neurons expressing mutant huntingtin implicating that BDNF modulation of striatal astrocyte function has therapeutic potential against neurodegeneration.

**Graphical abstract:**

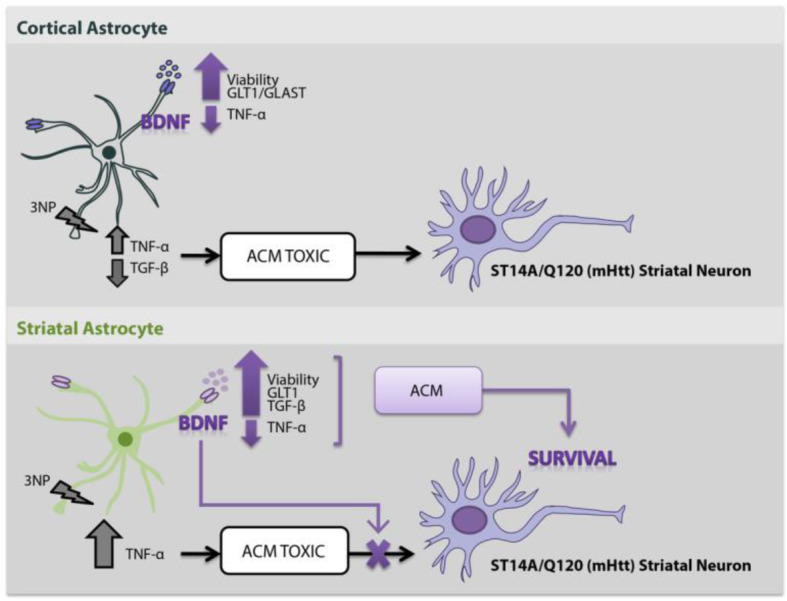

## Background

Huntington’s disease (HD) is an autosomal inherited neurodegenerative disorder in which the huntingtin gene undergoes an expansion of CAG triplets that produces the incorporation of polyglutamines to N-terminal region of the huntingtin protein (Htt). HD is characterized by the selective death of medium spiny GABAergic neurons in the striatum (basal ganglia) and, to a lesser extent, of cortical pyramidal neurons located mainly in layer V of the prefrontal cortex producing a progressive loss of motor and cognitive functions [1, 2]. Htt is expressed throughout the body, although its levels are higher in the central nervous system (CNS) [3]. Mutant huntingtin (mHtt) is believed to be toxic to neurons because it acquires an abnormal conformation forming inclusions in cytoplasm, nucleus, and also in axonal terminals as a consequence of failure in its degradation [4]. Other mechanisms such as modulation of transcription, proteasomal dysfunction, axonal transport, mitochondrial dysregulation, and excitotoxicity are also involved in HD pathogenesis. Brain-derived neurotrophic factor (BDNF) is a neurotrophin that promotes neuron survival and differentiation. BDNF was shown to exert neuroprotection against excitotoxicity involved in the degeneration of striatal neurons in HD [5]. Accumulated findings showed that BDNF’s mRNA and protein levels are significantly reduced in post-mortem samples from human HD cortex and striatum [6, 7]. Although this decrease is due to reduced BDNF transcription, it could also be caused by a defect in its transport [7, 8]. Depletion of BDNF in the striatum is a key pathogenic feature in HD, possibly responsible for the selective neuron vulnerability in this disease [4].

BDNF induces its effects through two types of receptors: it binds with high affinity to the tyrosine kinase receptor related to tropomyosin B (TrkB), and with low affinity to the p75 neurotrophin receptor, to which all neurotrophins could also be attached [9]. The binding of BDNF to TrkB induces its dimerization and autophosphorylation in the intracellular kinase domain, followed by activation of several intracellular signaling pathways. Two isoforms of TrkB generated by alternative splicing are abundantly expressed in the CNS: TrkB full length (TrkB-FL) and TrkB truncated 1 isoform (TrkB-T1) [10]. Through TrkB-FL, BDNF induces neuron differentiation, proliferation, and survival in the CNS [11–13]. Truncated TrkB-T1 lacks the kinase domain but contains a short isoform-specific cytoplasmic domain [14]. TrkB-T1 expression is the most abundant TrkB isoform in the adult rodent brain [15]. TrkB-T1 can oppose TrkB-FL function, BDNF binds TrkB-T1 instead of TrkB-FL which internalizes BDNF but does not autophosphorylate, thereby acting as a dominant negative receptor. However, TrkB-T1 has also been shown to induce neurite outgrowth and to regulate changes in the cytoskeleton [16]. Another study concluded that BDNF, through TrkB-T1, can activate signaling pathways such as extracellular signal-regulated kinase (ERK) and Akt, inducing neural stem cell proliferation [17]. We also demonstrated recently that TrkB-T1 is the major isoform expressed by astrocytes and that BDNF can induce protection from serum deprivation and 3-nitropropionic acid (3NP)-induced cell death through this receptor [18]. Activation of TrkB-T1 by BDNF was also proved recently to induce cortical astrocyte morphogenesis [19].

Astrocytes, the most numerous cell type in the CNS, have wide-ranging functions helping to maintain brain homeostasis. These cells are also activated against tissue damage or pathogens and are capable of producing a wide variety of pro-inflammatory mediators such as cytokines and chemokines. Neuroinflammation produced by the sustained activation of astrocytes over time is considered an important component in the development of neurodegenerative diseases [20]. Astrocytes can also play an important role in different stages of neurodegeneration that contribute to neuronal death. Marked astrogliosis was evidenced in HD patients and HD animal models [1, 21, 22]. Astrocytes maintain physiological levels of glutamate in the extracellular space. Accumulation of glutamate in the synaptic space produces neuron excitation; this accumulation was reported in several neurodegenerative diseases such as HD [23]. Since glutamate cannot be metabolized at the extracellular level, it has to be removed from the synaptic space. Thus, astrocytes have specific glutamate transporters: excitatory amino acid transporter 2 or, in the rat, glutamate transporter 1 (GLT1) and excitatory amino acid transporter 1 or, in the rat, glutamate aspartate transporter 1 (GLAST), both of these primarily responsible for glutamate uptake in the brain [24]. Also, research is beginning to address the heterogeneity of astrocytes. Evidence shows significant heterogeneity in many aspects of astrocytes, including morphology, gene expression profile, physiological properties, and function [25]. However, few studies have examined the role of astrocytes from different regions in HD. Very recently, a detailed study using transcriptomics and proteomics investigated the differences between control striatal astrocytes and HD striatal astrocytes, finding early dysfunction of astrocytes in two murine models of HD [26]. However, no data exists yet comparing different brain region astrocytes in HD.

Mitochondrial dysfunction is involved in the pathogenesis of HD. Systemic injection of 3NP, an irreversible inhibitor of mitochondrial complex II succinate dehydrogenase located in the inner membrane of the mitochondria, induces symptoms and mitochondrial dysfunction similar to those in HD patients [27]. ST14A cells are derived from embryonic rat striatal neurons with features of medium-sized spiny neurons [28, 29]. ST14A/Q15 (Q15) cells express the N-terminal fragment of human Htt with a 15 glutamine region (15 CAG repeats) and ST14A/Q120 (Q120) cells express the N-terminal fragment of human mHtt with a 120 glutamine region (120 CAG repeats). Q120 cells expressing mHtt fragments are more susceptible to death by 3NP than Q15 cells [30]. We have already proved that 3NP decreases viability in both cell lines in a dose-dependent manner [31]. We have previously demonstrated that BDNF decreases astrocyte death and oxidative stress induced by 3NP through TrkB-T1 receptor [18]. Since the most affected region in HD is the striatum, and then the cortex, we explored differences between astrocytes from these two areas. The aim of this study was to further investigate the mechanisms of BDNF protection and 3NP effect in cortical and striatal astrocytes using a cellular model of HD.

## Methods

### Reagents

TrkB inhibitor (ANA-12, N-[2-[[(Hexahydro-2-oxo-1H-azepin-3-yl)amino]carbonyl]phenyl]-benzo[b]thiophene-2-carboxamide Cat#SML0209) and 3-nitropropionic acid (3NP, Cat#N22908) 3-[4,5-dimethylthiazol-2-yl]-2,5-diphenyltetrazolium bromide (MTT, Cat#M5655) were obtained from Sigma-Aldrich Argentina. Ultrapure bacterial lipopolysaccharide (LPS) (*Escherichia* coli, 0111:B4, Cat#tlrl-3pelps) was from Invivogen (USA); BDNF was purchased from Alomone (Cat#B-250). Culture media and supplements were acquired from Invitrogen Argentina. All other media and supplements were obtained from Sigma-Aldrich Argentina, unless otherwise specified.

### Animals

Wistar rats (RRID:RGD_13508588) were housed in a temperature controlled facility at 25 °C on a 12 h light/12 h dark cycle with access to lab chow and water ad libitum. We bred these rats in our facility with one male and two females per cage. All animal care and experimental procedures were approved by the Institutional Animal Care and Use Committee of the School of Medicine of the University of Buenos Aires (EXP-UBA0049923/2015), in line with the National Institutes of Health Guidelines for the Care and Use of Laboratory Animals.

### Cell culture

Rat cerebral cortices or the striatum from 1- to 2-day-old Wistar rat pups were dissected and astrocytes were prepared as previously described [18]. Cells were seeded and maintained in Dulbecco’s modified eagle medium (DMEM)/F-12 medium containing 10% fetal bovine serum, 50 μg/ml streptomycin-50 U penicillin in 75 cm^2^ poly-l-lysine coated (10 μg/ml, Cat#P1524 Sigma-Aldrich) culture flasks at 37 °C in 5% CO_2_. Cell culture medium was changed twice a week. Astrocytes were further purified by shaking at 200 rpm overnight at 33 °C, trypsinized, and subcultured. After 3 days of stabilization, astrocytes were incubated with the drugs in DMEM containing 6 mM l-glutamine and 50 μg/ml of streptomycin-50 U penicillin. Cultures were routinely 95% astrocytes, as assessed by glial fibrillary acidic protein (GFAP, Millipore Cat#MAB360,RRID:AB_11212597) immunostaining. 3NP was dissolved in phosphate-buffered saline (PBS) and pH was adjusted to 7.4 with NaOH.

Immortalized striatal rat embryonic neurons which express a fragment of the N-terminal portion of normal human Htt (residues 1–548) with 15 glutamines or CAG repeats ST14A/Q15 (Q15) (Cat#CH00066) or expressing human mHtt with 120 glutamines or CAG repeats ST14A/Q120 (Q120) (Cat#CH01137) were purchased from Coriell Institute HD Community Biorepository. ST14A cells have medium sized spiny neuron features [28, 29]. Cells were grown in DMEM (Cat#12100-046) supplemented with 10% heat inactivated fetal bovine serum (Cat#NTC-1000 Natocor, Argentina), 2 mM l-glutamine (Cat#25030-081), and 100 μg/ml streptomycin-100 U penicillin (Cat#15140-122) and kept at 33 °C in 5% CO_2_. Q15 and Q120 cells behave very similarly in normal growth conditions until day 4 when Q120 cell number decreases, probably due to cell death [31]. Thus, all experiments performed were run at 1 to 3 days after subcultivation.

### Preparation of astrocyte-conditioned medium

Cortical and striatal astrocytes (8 × 10^4^ cells) were plated into 6-well plates and grown in DMEM containing 10% fetal bovine serum for 72 h. To prepare each astrocyte-conditioned medium (ACM), each group of astrocytes was incubated for 24 h in control conditions DMEM without serum (ACM-CTRL) or plus BDNF 50 ng/ml (ACM-BDNF), or with 3NP 15 mM (ACM-3NP) or 3NP + BDNF (ACM-3NP + BDNF). Then each ACM was collected, centrifuged at 2000 rpm for 10 min to remove cellular debris, and the supernatants stored at − 80 °C until use. When necessary, just before incubating ACM with ST14A neurons, we added 3NP 10 mM to the ACM, or in some experiments we added ANA-12 (1 μM) and 3NP to the ACM.

### Immunofluorescent identification of GFAP and ALDH1L1 in astrocytes

GFAP and aldehyde dehydrogenase 1 family member L1 (ALDH1L1) expression in astrocytes were evaluated by indirect immunofluorescence. Astrocytes were fixed with 0.5 ml 4% paraformaldehyde in PBS for 30 min at 4 °C. After rinsing with PBS, cells were incubated with 10% normal goat serum in PBS with 0.1% Triton X-100 for 60 min. Then, slides were incubated overnight at 4 °C with mouse anti-GFAP antibody (1:400, Millipore Cat# MAB360, RRID:AB_11212597) or anti-ADLH1L1 (1:50, Santa Cruz Biotechnology, Cat# sc-100497, RRID:AB_2224180) in PBS with 0.1% Triton X-100 and 1% normal goat serum. After rinsing, slides were incubated for 1 h with goat anti-mouse Alexa 488 (1:800, Jackson ImmunoResearch Labs Cat# 115-545-003, RRID:AB_2338840). Slides were mounted with mounting medium containing DAPI (Abcam) and visualized in a fluorescence microscope Axiophot (Carl Zeiss, Germany). Negative control slides were incubated with normal goat serum instead of primary antibody. Acquisition was performed with a × 40 objective. Fluorescence was determined using ImageJ software and was normalized to the number of cells.

### Cell viability assays

Trypan blue exclusion assay: Astrocytes or ST14A cells (2 × 10^5^ cells) were seeded onto 24-well plates and after incubation cells were washed with PBS and trypsinized. Pelleted cells were resuspended in DMEM + 10% fetal bovine serum and 10 μl of cell suspension were stained with 10 μl of Trypan blue. Viable cells that excluded Trypan blue dye were counted in a Neubauer chamber, and cell viability was expressed as number of live cells/ml. Each condition was tested in triplicate in every experiment.

MTT assay: Astrocytes (4 × 10^4^ cells) were seeded onto 96-well plates and after treatment cells were washed with Krebs buffer and incubated for 3 h in 100 μl Krebs buffer plus 50 μg of MTT reagent dissolved in 10 μl of PBS at 37 °C. The developed crystals were dissolved in 100 μl 0.04 N HCl in isopropanol and optical density was read in a microplate spectrophotometer al 595 nm.

### Western blot analysis

Astrocytes (8–10 × 10^5^ cells) and ST14A cells were lysed and proteins obtained as previously described [32]. Protein concentration of samples was determined by the Bradford assay. Then, 30–40 μg of proteins were size-fractionated in an SDS-PAGE and electrotransferred to a polyvinylidene difluoride membrane for 1 h at 4 °C or overnight at room temperature for TrkB. Blots were blocked for 1 h in 5% or 1% non-fat dry milk-TBS-0.1% Tween 20 and incubated overnight with the appropriate antibody. Anti-phosphorylated ERK (pERK, 1:200, Cell Signaling Cat# 4376 RRID:AB_331772), GLT1 (1:1000, Santa Cruz Biotechnology Cat# sc-365634,RRID:AB_10844832), GLAST (1:1000 Santa Cruz Biotechnology Cat# sc-515839), or TrkB (1:500 Millipore Cat# 07-225RRID: AB_310445) antibodies were incubated in 5% or 1% (for GLAST) non-fat dry milk in TBS-0.1% Tween 20. After washing, blots were incubated with the respective biotinylated HRP conjugated secondary antibody (Abcam) for 1 h. Immunoreactivity was detected by enhanced chemiluminescence kit (Biolumina, PBL, Argentina). After stripping, blots were incubated with total ERK (1:1000 Santa Cruz Biotechnology Cat# sc-135900, RRID:AB_2141283) or GAPDH (1:10000 Santa Cruz Biotechnology Cat# sc-25778 RRID: AB_10167668) antibodies, diluted in 5% non-fat dry milk-TBS-0.1% Tween 20 at 4 °C. Blots were analyzed using SCION Image software. Data were normalized to total ERK or GAPDH and values expressed as increments relative to the respective controls.

### Cytokine release

Astrocytes (4 × 10^4^ cells) were seeded onto 96-wellplates and after 24-h incubation, culture media was immediately frozen at − 80°C until use. Tumour necrosis factor-α (TNF-α) release was assessed by ELISA using a commercial kit (BD Biosciences, Cat#555268 San Diego, USA). Assays were performed following the manufacturer’s instructions. Cytokine values (pg/ml) were normalized to the viability values obtained by the MTT assay to account for variations in cell numbers caused by the different treatments. For determination of transforming growth factor-β (TGF-β) release, astrocytes (2 × 10^5^ cells) were seeded onto 24-well plates and after 24-h incubation, culture media was immediately frozen at − 80 °C until use. TGF-β release was assessed by ELISA using a commercial kit (Cat#BMS249-4 Invitrogen Argentina). Cytokine values (pg/ml) were normalized to the viability values obtained by the Trypan blue exclusion assay to account for variations in cell numbers caused by the different treatments.

### Statistical analysis

Data were expressed as mean ± SEM and analyzed by Student’s *t* test, one-way or two-way ANOVA, followed by Bonferroni multiple comparisons test using GraphPad Prism 8 software. Normal distribution in every group was tested using Anderson-Darling, D’Agostino-Pearson, Shapiro-Wild, and Kolmogorov-Smirnov tests and equality of variances was tested with Bartlett and Brown-Forsythe tests (ANOVA assumptions). Differences with a *p* value < 0.05 were considered statistically significant. Experiments were performed at least three times and the number of experiments performed is specified in each figure legend.

## Results

### Characterization of region-specific astrocytes

**Fig. 1:**
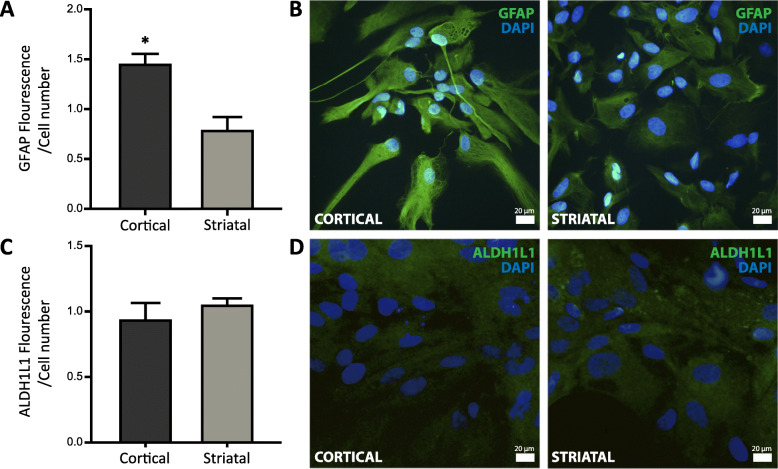
Characterization of region-specific astrocytes. **a** GFAP expression in cortical and striatal astrocytes was assessed by immunocytochemistry and normalized to the number of cells. Values represent the mean ± SEM of *n* = 3 independent experiments of GFAP fluorescence/cell determined by ImageJ software. Differences between two groups were analyzed by Student’s *t* test (**p < 0.01 vs. striatal astrocytes). **b** Representative merged images showing in green GFAP-positive cortical (left) and striatal (right) astrocytes and in blue their nuclei stained with DAPI. **c** ALDH1L1 expression in cortical and striatal astrocytes was assessed by immunocytochemistry and normalized to the number of cells. Values represent the mean ± SEM of *n* = 3 independent experiments of ALDH1L1 fluorescence/cell determined by ImageJ software. Differences between two groups were analyzed by Student’s *t* test. **d** Representative merged images showing in green cortical (left) and striatal (right) astrocytes positive for ALDH1L1 and in blue their nuclei stained with DAPI

We evaluated two specific markers of astrocytes: GFAP and ALDH1L1 in rat astrocyte cultures derived from cortical and striatal regions. Their expression was determined by immunocytochemistry. We found that GFAP expression in cortical astrocytes was higher than in striatal astrocytes (Fig. 1a, b). Regarding expression of ALDH1L1, we found no changes between astrocytes from the two areas studied (Fig. 1c, d).

### BDNF protects cortical and striatal astrocytes from 3NP-induced death.

**Fig. 2:**
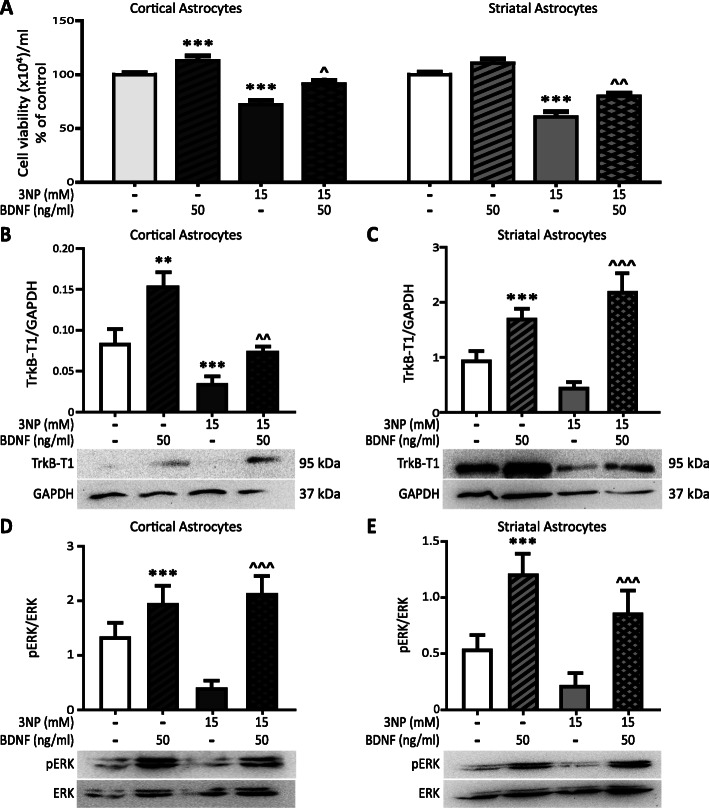
BDNF protects cortical and striatal astrocytes from 3NP-induced death. **a** Cell viability of cortical and striatal astrocytes treated for 24 h with 3NP 15 mM with or without BDNF 50 ng/ml was evaluated by Trypan blue exclusion assay. Values are the mean number of viable cells/ml ± SEM of *n* = 4 independent experiments for cortical and striatal astrocytes. The effect of both factors BDNF and 3NP was analyzed by two-way ANOVA: ***p < 0.001 vs. control and ^*p* < 0.05 and ^^*p* < 0.01 vs. 3NP. Cortical (**b**) and striatal (**c**) astrocytes were incubated for 24 h with 3NP 15 mM with or without BDNF 50 ng/ml and TrkB-T1 protein levels in total cell protein extracts were determined by Western blot. Values represent arbitrary units of TrkB-T1/GAPDH ratio ± SEM of *n* = 4 independent experiments for cortical and striatal astrocytes. The effect of both factors BDNF and 3NP was analyzed by two-way ANOVA: **p < 0.01 and ***p < 0.001 vs. control, ^^*p* < 0.01 and ^^^*p* < 0.001 vs. 3NP group. Cortical (**d**) and striatal (**e**) astrocytes were incubated for 30 min with 3NP 15 mM and with or without BDNF 50 ng/ml and pERK/total ERK protein levels in total cell protein extracts were determined by Western blot. Values represent arbitrary units of the pERK/total ERK ratio ± SEM of *n* = 5 independent experiments for cortical and striatal astrocytes. The effect of both factors BDNF and 3NP was analyzed by two-way ANOVA: ***p < 0.001 vs. control, ^^^*p* < 0.001 vs. 3NP group

We previously demonstrated that BDNF protects whole brain-derived astrocytes against 3NP-induced death [18]. To investigate the effect of BDNF on 3NP-induced death of cortical and striatal astrocytes, cells were treated with or without 3NP (15 mM) and/or BDNF (50 ng/ml) and viability was determined after 24 h. BDNF alone significantly increased viability in cortical astrocytes (Fig. 2a). 3NP decreased the number of viable cells in cortical and striatal astrocytes, but when co-incubated with BDNF, astrocyte viability increased in both populations (Fig. 2a). Next, we determined TrkB expression on cortical and striatal astrocytes. As we showed previously, astrocytes in culture mainly express TrkB-T1 protein [18]. Therefore, we measured TrkB-T1 protein expression by Western blot analysis. In both astrocyte populations, BDNF increased protein expression of TrkB-T1 regardless of 3NP presence (Fig. 2b, c). 3NP significantly decreased protein levels of this receptor only in cortical astrocytes (Fig. 2b). Also, since we already proved that BDNF induces ERK activation in whole brain astrocytes [18], we verified if this is also true for striatal and cortical astrocytes separately. We confirmed that BDNF increased levels of pERK in cortical and striatal astrocytes, and we found that, although showing a tendency to decrease, 3NP did not significantly modify basal pERK activation in astrocytes (Fig. 2d, e). When treated with 3NP + BDNF, activation of pERK was still increased in both astrocyte populations (Fig. 2d, e). Therefore, cortical and striatal astrocytes expressed functional TrkB-T1 and BDNF protected both types of astrocytes from 3NP toxicity.

### BDNF increases protein expression of glutamate transporters

**Fig. 3:**
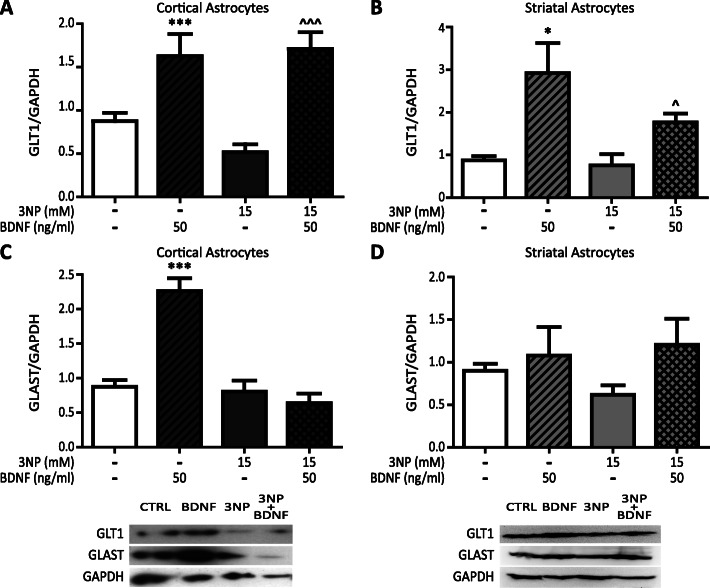
BDNF increases protein expression of glutamate transporters. Cortical (**a**) and striatal (**b**) astrocytes were incubated for 24 h with 3NP 15 mM with or without BDNF 50 ng/ml and GLT1 protein levels in total cell protein extracts were determined by Western blot. Values represent arbitrary units of the GLT1/GAPDH ratio ± SEM of *n* = 5 independent experiments for cortical and 4 independent experiments for striatal astrocytes. The effect of both factors BDNF and 3NP was analyzed by two-way ANOVA *p < 0.05 and ***p < 0.001 vs. control group ^*p* < 0.05 and ^^^*p* < 0.001 vs. 3NP group. Cortical (**c**) and striatal (**d**) astrocytes were incubated for 24 h with 3NP 15 mM with or without BDNF 50 ng/ml and GLAST protein levels in total cell proteins extracts were determined by Western blot. The values represent arbitrary units of the GLAST/GAPDH ratio ± SEM of *n* = 4 independent experiments for cortical and *n* = 5 independent experiments for striatal astrocytes. The effect of both factors BDNF and 3NP was analyzed by two-way ANOVA. ***p < 0.001 vs. control group

To control glutamate in particular, astrocytes have specific transporters: GLT1 and GLAST. Since in HD the expression of GLT1 decreases [22], we evaluated protein expression of these transporters in both astrocyte populations. We found that BDNF increased expression of GLT1 in astrocytes from cortex and striatum, even in the presence of 3NP (Fig. 3a, b). As for the GLAST transporter, we found that BDNF increased GLAST expression only in cortical astrocytes (Fig. 3c), whereas in striatal astrocytes, neither BDNF nor 3NP changed the expression of the transporter (Fig. 3d).

### BDNF modifies astrocyte cytokine release

**Fig. 4:**
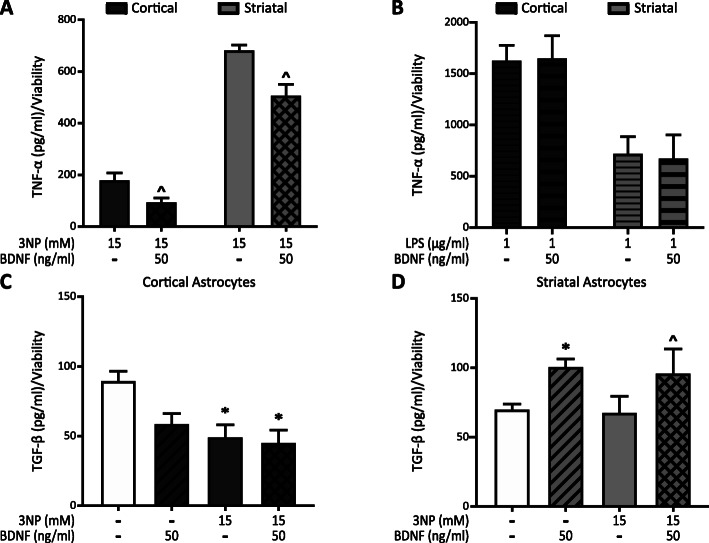
BDNF modifies astrocyte cytokine release. Cortical and striatal astrocytes were treated for 24 h with 3NP 15 mM with or without BDNF 50 ng/ml (**a**) or with LPS 1 μg/ml with or without BDNF 50 ng/ml (**b**). TNF-α release into the culture supernatant was assessed by ELISA and normalized to viability values obtained with the MTT assay for each experimental group. Data are the mean ± SEM of *n* = 3 independent experiments. Differences between two groups were analyzed by Student’s *t* test. ^*p* < 0.05 vs. 3NP group. Cortical (**c**) and striatal (**d**) astrocytes were treated for 24 h with 3NP 15 mM with or without BDNF 50 ng/ml. TGF-β release into the culture supernatant was assessed by ELISA and normalized to viability values obtained by Trypan blue exclusion assay for each experimental group. Data are the mean ± SEM of *n* = 3 independent experiments. The effect of both factors BDNF and 3NP was analyzed by two-way ANOVA. *p < 0.05 vs. control group ^*p* < 0.05 vs. 3NP group

One possible mechanism for BDNF protection in astrocytes could be reduction of the inflammatory response. In order to evaluate the effect of BDNF on 3NP-induced cytokine release, we determined levels of the pro-inflammatory cytokine TNF-α and the anti-inflammatory cytokine TGF-β in the astrocyte supernatant upon 3NP treatment. TNF-α levels are increased in the striatum of animals treated with 3NP [33]. Concordantly, 3NP-induced release of TNF-α to the culture medium (Fig. 4a), not detected in control or BDNF groups from both astrocyte populations. Surprisingly, astrocytes from the striatum released 3 times higher levels of TNF-α than cortical astrocytes, and BDNF decreased TNF-α release induced by 3NP in both populations (Fig. 4a). To verify that cortical astrocytes are indeed capable of releasing high levels of TNF-α, we tested the effect of bacterial lipopolysaccharide (LPS), a potent pro-inflammatory stimulus, in astrocytes [34]. LPS (1 μg/ml) induced a remarkable increase of TNF-α levels secreted by cortical astrocytes and the presence of BDNF did not change this effect (Fig. 4b). However, striatal astrocytes release lower levels of TNF-α in response to LPS than cortical astrocytes, similar to 3NP-induced levels (Fig. 4a, b). BDNF did not modify LPS effect on TNF-α release in striatal astrocytes. Data suggest that different inflammatory stimuli caused different astrocyte responses and that BDNF action is clearly specific to inflammation induced by 3NP. TGF-β is a key molecule in brain development and brain homeostasis. TGF-β is decreased in HD patients [35] and is known to have an anti-inflammatory role in the brain [36]. Therefore, we investigated whether astrocytes from cortex and striatum could release TGF-β in response to 3NP or BDNF. We found that 3NP, alone or in the presence of BDNF, decreased TGF-β release, whereas BDNF per se did not modify the release of TGF-β from cortical astrocytes (Fig. 4c). In striatal astrocytes, 3NP had no effect on TGF-β release and BDNF increased TGF-β release of striatal astrocytes regardless of the presence of 3NP (Fig. 4d). Considered altogether, our data indicate that cortical and striatal astrocytes responded differently to 3NP action. Striatal astrocytes released more TNF-α and cortical astrocytes less TGF-β in response to 3NP. Nevertheless, BDNF exerted an anti-inflammatory effect on both populations.

### BDNF protects ST14A/Q120 neurons from 3NP-induced cell death.

**Fig. 5:**
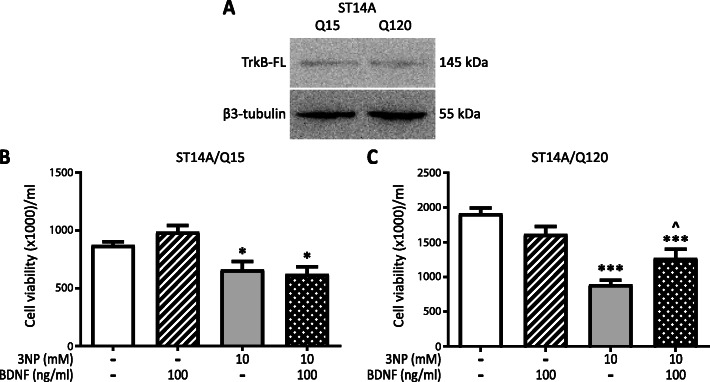
BDNF protects ST14A/Q120 neurons from 3NP-induced cell death. **a** TrkB-FL and β3-tubulin protein levels in total cell protein extracts of Q15 or Q120 cells in control conditions were determined by Western blot. Image shows one representative blot out of two. Q15 (**b**) and Q120 (**c**) cells were incubated with 3NP 10 mM with or without BDNF 100 ng/ml for 24 h and cell viability was evaluated by Trypan blue exclusion assay. Values are the mean ± SEM of viable cells/ml of *n* = 3 independent experiments. The effect of both factors BDNF and 3NP was analyzed by two-way ANOVA. *p < 0.05, ***p < 0.001 vs. control group and ^*p* < 0.05 vs. 3NP group

Given that BDNF is required for striatal neuron survival [37], we determined BDNF effect on striatal neurons. For this purpose, we used rat striatal embryonic neuronal cell line ST14A expressing normal human Htt with 15 CAG repeats (Q15) and ST14A cells expressing human mHtt with 120 CAG repeats (Q120) [31], as a cellular model of HD. First, we confirmed that both cell lines expressed TrkB-FL (Fig. 5a). Then we tested whether BDNF could protect Q15 and Q120 cells from 3NP-induced death. BDNF per se did not modify Q15 or Q120 cell viability, but BDNF partially blocked 3NP-induced cell death in Q120 neurons (Fig. 5c). Surprisingly, BDNF did not inhibit death of Q15 cells induced by 3NP (Fig. 5b). These results suggest that BDNF selectively protected neurons expressing mHtt.

### Differential effect of astrocyte-conditioned medium from BDNF treated astrocytes on neuron viability

**Fig. 6:**
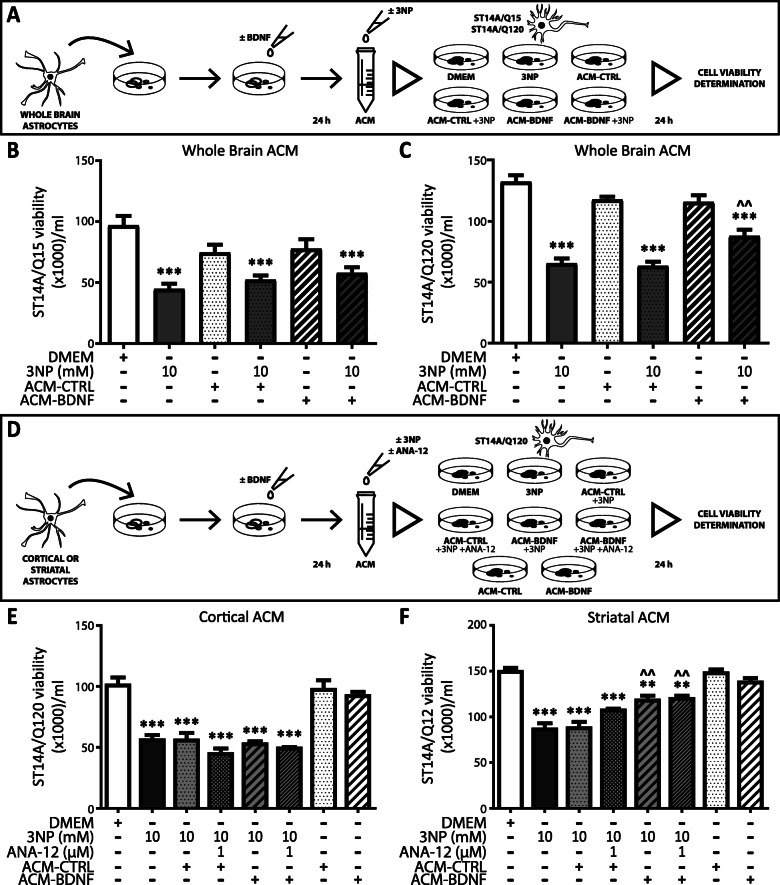
Regional difference in ACM from BDNF-treated astrocytes effect on neuron viability. **a** Time schedule of ACM collection and treatment. ACM was prepared from astrocytes treated for 24 h with BDNF 50 ng/ml (ACM-BDNF) or without (ACM-CTRL). Q15 and Q120 cells were treated for 24 h with 3NP (10 mM) added to each ACM. Effects of ACM on viability of Q15 (**b**) or Q120 (**c**) cells were evaluated by Trypan blue exclusion assay. Data represent the mean number of viable cells/ml ± SEM of *n* = 4 experiments. Differences between all groups were analyzed by One-way ANOVA followed by Bonferroni’s multiple comparison post-test. ***p < 0.001 vs. DMEM and ^^*p* < 0.01 vs. ACM-CTRL + 3NP. **d** Time schedule of ACM collection and treatment. ACM was prepared from astrocytes treated for 24 h with BDNF 50 ng/ml (ACM-BDNF) or without (ACM-CTRL). Q15 and Q120 cells were treated for 24 h with 3NP (10 mM) and if necessary, ANA-12 (1 μM) was added to ACM. Q120 cells were treated with ACM from cortical (**e**) or striatal (**f**) astrocytes. Values represent the mean number of viable cells ml ± SEM of *n* = 4 experiments. Differences between all groups were analyzed by one-way ANOVA followed by Bonferroni’s multiple comparison post-test. **p < 0.01, ***p < 0.001 vs. control and ^^*p* < 0.01 vs. ACM-CTRL + 3NP

**Fig. 7:**
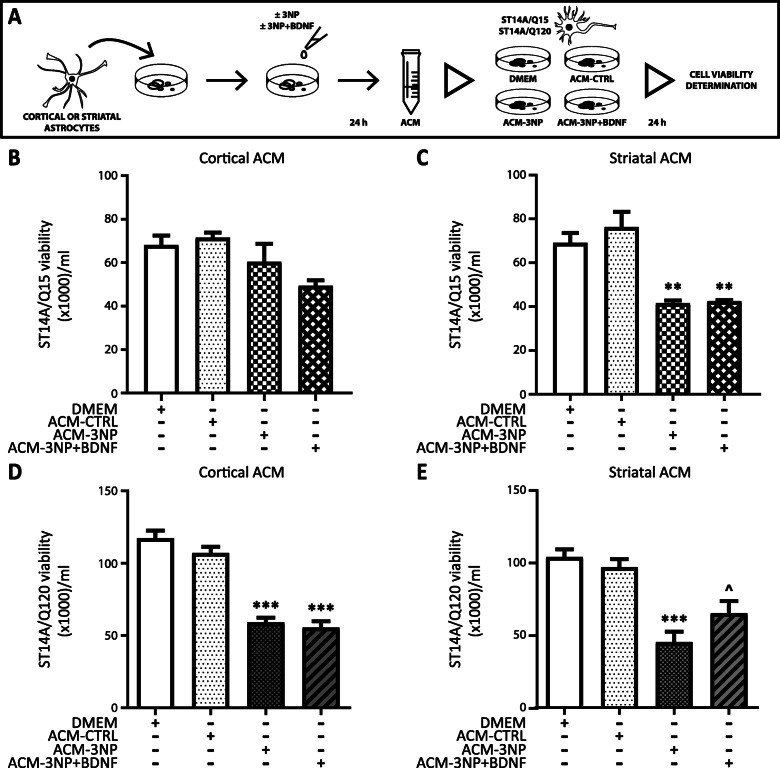
Regional difference in ACM from 3NP+BDNF-treated astrocytes on neuron viability. **a** Time schedule of ACM collection and treatment. Cortical or striatal astrocytes were treated for 24 h with DMEM (ACM-CTRL), or 3NP 15 mM (ACM-3NP), or 3NP 15 mM and BDNF 50 ng/ml (ACM-3NP + BDNF). Q15 and Q120 cells were treated for 24 h with those ACM. Q15 cells were incubated for 24 h in the presence or absence of ACM from cortical (**b**) and striatal (**c**) astrocytes. Q120 cells were incubated for 24 h in the presence or absence of ACM from cortical (**d**) and striatal (**e**) astrocytes. Cell viability was evaluated by Trypan blue exclusion assay. Data show mean number of viable cells/ml ± SEM of *n* = 4 experiments. Differences between all groups were analyzed by one-way ANOVA followed by Bonferroni´s multiple comparison post-test. **p < 0.01, ***p < 0.001 vs. control and ^*p* < 0.05 vs. ACM-3NP

Our previous results showed that conditioned media from BDNF-treated astrocytes protects PC12 cells from death induced by 3NP [18]. Since BNDF increases astrocyte viability and function, we wondered if BDNF-treated astrocytes could have a protective effect in a cellular model of HD. Therefore, we evaluated whether astrocyte-conditioned media (ACM) could induce neuroprotection in Q15 and Q120 striatal neurons. First, we treated whole brain astrocytes without (ACM-CTRL) or with BDNF (ACM-BDNF) for 24 h and then Q15 and Q120 cells were incubated with ACM from whole brain astrocytes in the presence or absence of 3NP for 24 h (Fig. 6a). In this set of experiments, we discovered that ACM-CTRL and ACM-BDNF did not modify viability of Q15 cells in any condition tested (Fig. 6b). Conversely, although ACM per se had no effect on Q120 cell viability, only ACM-BDNF partially protected Q120 neurons from 3NP-induced death (Fig. 6c). Data indicate that the effect of ACM-BDNF was selective to cells expressing mHtt. To further examine the neuroprotective effect of ACM-BDNF, we treated cortical and striatal astrocytes without (ACM-CTRL) or with BDNF (ACM-BDNF) for 24 h and used these ACM to incubate Q120 cells, in the presence or absence of 3NP, for 24 h (Fig. 6d). Also, we added ANA-12, an antagonist of the TrkB receptor, to the ACM-BDNF in order to block the action exerted by any remnant of BDNF in the ACM (Fig. 6d). We found that ACM-CTRL and ACM-BDNF from cortical astrocytes either with or without addition of ANA-12 did not protect Q120 cells from 3NP-induced cell death (Fig. 6e). ACM-CTRL from striatal astrocytes did not modify Q120 cell viability but ACM-BDNF from striatal astrocytes increased Q120 viability in the presence of 3NP (Fig. 6f). Since addition of ANA-12 in ACM-BDNF did not modify the effect of ACM-BDNF on Q120 neuron viability, we confirmed that the effect observed was exclusively due to ACM-BDNF action and not to any remnant of BDNF in the ACM. These results strengthen the idea that only striatal astrocytes treated with BDNF released neuroprotective factors able to prevent 3NP toxicity in neurons expressing mHtt. We also tested the effect of ACM from cortical or striatal astrocytes treated for 24 h with 3NP (ACM-3NP) or with 3NP and BDNF (ACM-3NP + BDNF) on Q15 and Q120 cell survival (Fig. 7a). None of the ACM from cortical astrocytes altered viability of Q15 cells (Fig. 7b). Remarkably, ACM-3NP and ACM-3NP + BDNF from striatal astrocytes diminished Q15 cell viability in a similar way (Fig. 7c), indicating that ACM-3NP from striatal astrocytes, regardless of the presence of BDNF, was more toxic than cortical ACM-3NP. Concerning Q120 cells, we found that cortical ACM-3NP and cortical ACM-3NP + BDNF induced their death (Fig. 7d), confirming that Q120 cells are more sensitive to toxic stimuli than Q15 cells. ACM-3NP from striatal astrocytes seemed slightly more effective than cortical ACM-3NP in inducing Q120 cell death (Fig. 7e). Nevertheless, ACM-3NP + BDNF had a less toxic effect since ACM-3NP + BDNF increased Q120 neuron viability compared to ACM-3NP (Fig. 7e). These results reinforce that only striatal astrocytes treated with BDNF release neuroprotective factors able to protect mHtt expressing neurons from 3NP toxicity.

## Discussion

Astrocytes have traditionally been viewed as simple homogenous cells providing support to neurons. We now recognize that astrocytes from different brain regions are heterogeneous [38]. Here, we characterized astrocytes form cortex and striatum, two areas involved in HD pathogenesis. Cortical astrocytes in culture express higher levels of GFAP than striatal astrocytes. Similar results for in vivo experiments assaying GFAP expression by proteomic and transcriptomic analysis showed that striatal astrocytes had lower levels of GFAP expression than hippocampal astrocytes [39]. Chai and collaborators also demonstrated that cortical astrocytes and hippocampal astrocytes were really similar when comparing RNA sequencing data but hippocampal and striatal astrocytes were shown to be different populations. Given that culture astrocytes are mechanically manipulated, they are likely to be reactive. However, as we showed in this work, control astrocytes did not secrete detectable levels of TNF-α, and they preserve the ability to be activated by 3NP or LPS, showing that they can react similarly to in vivo astrocytes. Our results indicate that cortical and striatal astrocytes in culture mirrored some differences and features of in vivo astrocytes.

Although numerous studies focus on BDNF effects on neuron development and survival, surprisingly little is known about BDNF effects on astrocytes. We already demonstrated that BDNF prevents apoptotic death of whole brain rat astrocytes induced by serum deprivation and 3NP stimuli [18]. 3NP is a toxin resembling HD mitochondrial dysfunction. It is widely used as an in vitro model of HD, causing striatal degeneration as it occurs in HD [27]. Moreover, 3NP toxicity induces the decrease of GFAP immunoreactivity by affecting the removal of glutamate from the synaptic cleft, cell survival, and reducing the release of trophic factors [40]. Here, we demonstrate that BDNF protects cortical and striatal astrocytes from 3NP-induced death, mainly through TrkB-T1. Although in striatal astrocytes protein levels of TrkB-T1 seem to be higher, both astrocyte populations can respond to BDNF. Also, BDNF increased its own receptor TrkB-T1 expression and signaling by activating ERK. We previously demonstrated ERK activation in whole brain astrocytes [18]. Thus, BDNF activates TrkB-T1 and ERK signaling, resulting in increased survival of both cortical and striatal astrocytes. This finding agrees with other studies where TrkB-T1 function participates in morphogenesis of cortical astrocytes [19] and where TrkB-T1 KO astrocytes demonstrated downregulation of migration and proliferation pathways [41].

Impairment of glutamate signaling or uptake is associated with many neurological disorders, including HD. Alteration of glutamate receptor distribution and signaling at cortico-striatal synapses and alteration of glutamate release contribute to onset of the disease [42]. Our results indicate that 3NP did not modify the expression of GLT1 or GLAST transporter, whereas BDNF markedly increased GLT1 expression in both cortical and striatal astrocytes. BDNF regulated GLAST expression differently since it was increased only in cortical astrocytes. Strong evidence shows progressive loss of GLT1 expression in HD mouse models [42]. Given that GLT1 has a predominant role in glutamate uptake, BDNF could diminish GLT1 loss in HD, thereby contributing to its beneficial effects in HD models. We have proved before that BDNF increases intracellular levels of glutathione, the main antioxidant molecule, which is formed by glutamate, and removes excess of reactive oxygen species [18]. We consider that increasing GLT1 contributed directly to maintenance of the cortical-striatal regulation of glutamate uptake and can also contribute to glutathione generation and avoidance of neuron dysfunction. Further in vivo studies are needed in order to reinforce astrocytes as therapeutic targets for ameliorating HD neuropathology.

Pro-inflammatory cytokines were reported to regulate glutamate transporters both in vivo and in vitro [43]. TNF-α, a well-known inhibitor of glutamate re-uptake, could also induce oxidative stress and neuron death [44]. TNF-α protein levels in the striatum are increased in an HD model induced by 3NP [33] and in a transgenic HD mouse model R6/2 [45]. Moreover, when post-mortem HD striatal tissue was evaluated, TNF-α striatal mRNA was higher in HD patients [46] and thus excessive TNF-α was proposed to be one mechanism of neurodegeneration [47]. Here, we found that 3NP stimulates release of TNF-α in both astrocyte populations, but striatal astrocytes have a greater response to 3NP than cortical astrocytes. Striatal astrocytes were even shown to have the highest levels of peroxide production and necrotic cell death rates compared to cortical and mesencephalic astroglia in response to 3NP [48], reinforcing the idea that striatal astrocytes are more susceptible to mitochondrial dysfunction by 3NP. More interestingly, BDNF decreased TNF-α release induced by 3NP, indicating that BDNF may have an underappreciated anti-inflammatory role in the brain. Regarding LPS, a strong pro-inflammatory stimulus, we found that astrocytes react oppositely compared to their reaction to 3NP. In this experiment, cortical astrocytes released greater amounts of TNF-α than striatal astrocytes, and BDNF had no effect on LPS action. Thus, BDNF action is specific to 3NP-induced inflammation. Data suggest that astrocyte heterogeneity must be considered in order to evaluate their anti-inflammatory response. TGF-β can be essential for many processes such as neurogenesis, synapse formation, gliogenesis, and angiogenesis [49]. Recently, interesting data show that TGF-β deficiency could affect loss of astrocyte glutamate transporters such as GLT1 or GLAST, and also decreased glutamate uptake [50]. Concordantly, TGF-β expression is decreased in HD patients [35]. In the present work, we found that astrocytes respond differently to 3NP insult. Although 3NP decreased the levels of TGF-β in cortical astrocytes, it did not modify TGF-β release in striatal astrocytes. In contrast, whereas BDNF per se did not modify TGF-β levels in cortical astrocytes, it increased TGF-β release selectively from striatal astrocytes. These results suggest that cortical astrocytes decreased TGF-β release in response to 3NP possibly affecting astrocyte inflammatory response. Striatal astrocytes can respond to BDNF by increasing TGF-β. Restoring TGF-β in striatal astrocytes could be beneficial in the context of neurodegenerative processes such as HD.

BDNF is a widely known protective neurotrophic factor: in in vivo models, transport of BDNF is markedly decreased in cortical neurons and over expression of BDNF rescues changes in neuron structures and function in HD mice model [51]. BDNF suppresses autophagy induced by 3NP [52], and prevents death by 3NP in rat cortical neurons [53]. In our work, 3NP effectively induced neuron death and BDNF protected Q120 cells expressing mHtt. Unexpectedly, BDNF did not prevent 3NP-induced death in Q15 cells. Similar results were reported by Martire et al. [54], where BDNF prevented NMDA toxicity selectively in Q120 but not in Q15 cells. Since both Q15 and Q120 neurons express TrkB-FL, BDNF failure to protect Q15 might involve defective TrkB signaling. It is also possible that this cell line is less susceptible to external stimuli than primary neurons, and that expression of mHtt, but not Htt, markedly increases susceptibility to damage.

Aside from astrocyte dysfunction, gain of function in astrocytes was also shown to contribute to the toxicity observed in neurodegenerative diseases [55]. A recent study on HD astrocyte transcriptome and proteome suggests that in HD models, striatal astrocytes lose their functions rather than acquire a toxic phenotype [26], although further studies are needed to univocally prove this hypothesis. On the other hand, many studies have provided strong evidence that astrocytes actively contribute to neuroprotection [56]. In fact, glial-conditioned medium is rich in antioxidants and neurotrophic factors that may help protect damaged neurons [57]. We previously demonstrated that ACM from BDNF-treated astrocytes protected PC12 neurons from 3NP toxicity [18]. Now, we show that ACM from BDNF-treated astrocytes exerted selective protection of mHtt expressing striatal neurons without affecting normal Htt expressing cells. Moreover, when differentiating cortical from striatal ACM, protection was exerted only by ACM from BDNF-treated striatal astrocytes. This was not due to remaining BDNF in the culture media because blocking it by adding ANA-12 to the ACM did not modify the effect of ACM-BDNF on Q120 cell viability. Concerning cortical ACM-3NP and striatal ACM-3NP, both induced Q120 cell death which could be due to TNF-α released by astrocytes in response to 3NP. Actually, excesses of TNF-α coactivate astrocytes resulting in neuron dysfunction and ultimately neuron death [58]. Only striatal ACM from 3NP + BDNF-treated astrocytes increased Q120 cell viability. Therefore, striatal ACM-BDNF, unlike cortical ACM-BDNF, contains soluble factors that exert neuroprotection. TGF-β might be one protective factor released by astrocytes upon BDNF stimulation, although further studies measuring other soluble factors such as growth factors, cytokines, and neuropeptides are needed to fully elucidate these mechanisms.

A number of studies suggest that heterogeneity of astrocytes must be considered in regard to neurodegenerative progression [59]. One study in particular recently evidenced the difference between striatal astrocytes and mesencephalic astrocytes [60]. Asanuma et al. (2019) demonstrated that striatal astrocytes promoted stronger neuroprotection than mesencephalic astrocytes against oxidative stress. They attributed this difference to the altered expression of genes induced in these brain areas and showed that striatal astrocytes upregulate antioxidant and detoxifying pathways compared to midbrain astrocytes.

Few studies have investigated the differences between astrocytes from different regions. Given the complexity of the CNS, only in the last decades have studies on glial responses in vivo began to be conducted. Primary cultured astrocytes have proved to be an important tool in elucidating glial physiology since 1980 [61]. Nevertheless, cultured astrocytes are not equal to in vivo astrocytes since for example they modify gene expression in the presence of fetal serum added to primary cultures [62]. This study shows that isolation of astrocytes by immunopanning technique yields astrocytes with a more similar pattern of gene expression to that of in vivo astrocytes than do traditional primary astrocyte cultures. However, ACM from classical astrocyte cultures exhibit the same protective effect on neuron survival than ACM from astrocytes isolated by immunopanning technique. Moreover, astrocytes isolated by immunopanning technique also increase synapse formation and function in a similar way as classically cultured astrocytes do. Thus, astrocytes change under culture conditions but they still perform functions that can help understand glial biology. We present here clear differences between cortical and striatal astrocytes. Although in vivo studies are needed to confirm this data, our results strongly suggest that striatal astrocytes, though more susceptible to HD-like toxic stimulus, exert neuroprotection in mHtt expressing neurons in response to BDNF.

## Conclusions

All the findings presented here point to a critical role for BDNF-treated striatal astrocytes in preventing striatal dysfunction and also in providing neuronal support in an HD-like context. Importantly, our results demonstrate that cortical and striatal astrocytes differed in their response to toxic and protective stimuli, thereby further proving their heterogeneity. Understanding astrocyte heterogeneity is paramount for unraveling their protective potential in neurodegenerative disorders.

## Data Availability

The datasets used and/or analyzed during the current study are available from the corresponding author on reasonable request.

## Abbreviations

ALDH1L1: Aldehyde dehydrogenase 1 family member L1
ACM: Astrocyte-conditioned medium
BDNF: Brain-derived neurotrophic factor
CNS: Central nervous system
DAPI: 4′,6-Diamido-2-phenylindole dihydrochloride
DMEM: Dulbecco’s modified eagle medium
ERK: Extracellular signal-regulated kinase
GFAP: Glial fibrillary acidic protein
GLAST: Glutamate aspartate transporter 1
GLT1: Glutamate transporter1
Htt: Huntingtin
mHtt: Mutant huntingtin
HD: Huntington’s disease
LPS: Bacterial lipopolysaccharide
MTT: 3-[4,5-Dimethylthiazol-2-yl]-2,5-diphenyltetrazolium bromide
3NP: 3-Nitropropionic acid
PBS: Phosphate-buffered saline
TNF-α: Tumour necrosis factor-α
TGF-β: Transforming growth factor-β
TrkB: Tyrosine kinase receptor related to tropomyosin B
TrkB-FL: TrkB-full length
TrkB-T1: TrkB-truncated
Q15: ST14A cells expressing normal human htt
Q120: ST14A cells expressing mutant human htt
